# Role of core-shell energetics on anti-Mackay, chiral stacking in AgCu nanoalloys and thermally induced transition to chiral stacking

**DOI:** 10.1038/s41598-020-60059-6

**Published:** 2020-02-24

**Authors:** Manoj Settem, Anand K. Kanjarla

**Affiliations:** Department of Metallurgical and Materials Engineering, Indian Institute of Technology Madras, Chennai, 600036 India

**Keywords:** Theory and computation, Materials science, Nanoparticles

## Abstract

In AgCu nanoalloys a size-dependent transition to the chiral stacking from the anti-Mackay stacking has been predicted previously. This trend is explained by considering the interplay between the core-shell energetics. Results indicate that the energy changes in the Ag shell alone is not sufficient to explain the stability of the chiral stacking and the energy changes in the Cu core also need to be considered. In addition to this, thermally induced transition to chiral stacking was observed at sizes where anti-Mackay stacking is energetically favourable. On transition to the chiral stacking, the Ag-Ag, Ag-Cu and Cu-Cu bond lengths change significantly. These observations are also applicable for AgCu nanoalloys with incomplete Ag shells.

## Introduction

Bimetallic nanoalloy systems exhibit novel properties due to synergistic effects between the constituent metals. Bimetallic nanoclusters and nanoparticles have been extensively studied as they afford the opportunity to tune their chemical and physical properties through composition and chemical ordering^1–3^. Along with the geometrical structure of a bimetallic nanocluster, due to the presence of two elements, different types of chemical ordering are possible: phase segregated and mixed. Depending on the bimetallic system, phase segregation can manifest as core-shell structure^4,5^ where one of the constituent element covers the other completely or two phases joined at an interface, usually referred to as "Janus” type arrangemen^6–8^. Similarly, mixed chemical ordering can be ordered (as is the case for bimetallic systems that form intermetallic phases)^9^ or disordered^10^.

It is essential to know the optimal structures as a function of the size and the composition in order to understand the properties of bimetallic nanoclusters. Finding the optimal structures involves exploring the potential energy surface (PES)^11^. Typically, methods based on genetic algorithm^12^ or Monte Carlo combined with energy minimization commonly referred to as "basin hopping” Monte Carlo^13^ are used to explore the PES and find the optimal structures using empirical atom-atom potentials such as the second moment approximation to tight binding (SMATB)^14–17^ potential or the embedded atom method (EAM)^18^ potential. Finally, the putative global minima and the lowest lying isomers belonging to the different structural motifs are singled out for relaxation using density functional theory (DFT). This provides a database of optimal structures which can be used to compare with the experimentally synthesized bimetallic nanoclusters.

Depending on the constituent atoms in a bimetallic nanoalloy, specific structural motifs can become energetically favourable. The anti-Mackay icosahedron becomes stable in alloy systems which have a tendency to phase segregate and have atomic size mismatch combined with low surface energy for the larger atoms^19–21^. AgCu, AgNi and AgCo are examples of such systems and share common features of phase segregation with Ag being the larger atom which has low surface energy compared to Cu, Ni and Co. In contrast, for pure (single element) metal nanoclusters, incomplete Mackay icosahedron is preferred. In these systems, Bochicchio *et al*.^20^ identified a new class of structural motif, the chiral icosahedron, which loses all its mirror symmetries but retains the rotational symmetries. Recent theoretical work^22^ on Au${}_{60}$ shell showed a similar transition to chiral structure. However, in contrast to AgCu where the Ag shell is stacked on the Cu core, Au${}_{60}$ is a hollow structure. Chiral decahedra have been found to be energetically favourable with respect to regular decahedra for Au${}_{122}$Co${}_{75}$, Ag${}_{102}$Cu${}_{54}$, and Ag${}_{172}$Cu${}_{146}$ although they are not the global minima^23^. Chirality at nanoscale is being studied actively and we point to reviews^24–26^ on the topic and the references therein for a comprehensive outlook.

**Figure 1:**
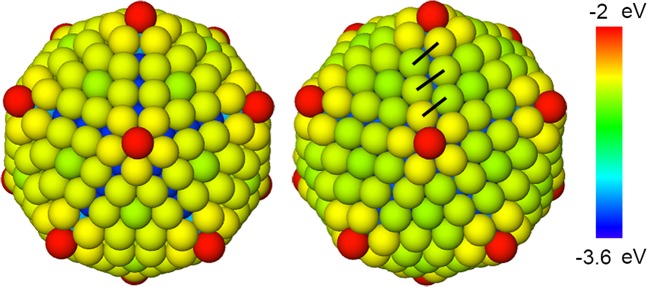
Anti-Mackay icosahedron and chiral icosahedron for Ag${}_{212}$Cu${}_{309}$ nanoalloy. The atoms are colored according to energy.

In the anti-Mackay icosahedron (Fig. 1), the triangular {111}-like planes on the surface have hcp-like stacking. We will refer to this arrangement of {111}-like planes as the anti-Mackay stacking. The chiral structure is obtained by a concerted rotation of the triangular {111}-like planes. We will refer to this arrangement of the {111}-like planes as the chiral stacking. Bochicchio *et al*.^20^ reported a size dependent transition from the anti-Mackay to the chiral structure. Stability of the chiral structures was attributed to the formation of new Ag-Ag bonds (black lines in Fig. 1) which become less stretched at larger sizes. Our results show that in addition to this, the interplay between core-shell energetics should be considered to understand the size-dependent stability of the anti-Mackay or the chiral stacking.

In the AgCu system, at lower sizes of 127 and 279, the anti-Mackay structures have lower energy compared to the chiral structures. Beginning from the size 521, the chiral structure becomes energetically favourable^20^. In the current work, we study the role of core-shell energetics in anti-Mackay and chiral icosahedra in AgCu nanoalloys in relation to the the size-dependent transition to the chiral structure using molecular dynamics simulations. We also report a thermally induced transition to the chiral stacking in the structures for which the anti-Mackay stacking is energetically favourable.

## Results and Discussion

In the following simulations, the interactions between Ag-Ag, Cu-Cu and Ag-Cu were modelled using an alloy EAM potential developed by Williams *et al*.^27^ The simulations were carried out using LAMMPS^28^ package and OVITO^29^ was used for structure visualization and analysis. In the following sections, when we we refer to sizes 127, 279, 521, and 873, it is implied that we are referring to the following nanoalloys Ag${}_{72}$Cu${}_{55}$, Ag${}_{132}$Cu${}_{147}$, Ag${}_{212}$Cu${}_{309}$, and Ag${}_{312}$Cu${}_{561}$ respectively and we use them interchangeably.

### Core-shell energetics

In order to understand the stability of the anti-Mackay and the chiral stacking, it proves to be useful to consider the energy of the Ag shell and the Cu core separately. Such a separation into distinct components (shell, core) is possible due to the use of atom-atom potentials. The EAM atom-atom potential used in the current work predicts the same stability order as predicted by Bochicchio *et al*.^20^ using SMATB potential i.e. the anti-Mackay icosahedron is lower in energy at the sizes 127, 279 and the chiral icosahedron is lower in energy at the sizes 521, 873. However, in variance with the SMATB potential, chiral icosahedron at 279 is not a stable local minimum under EAM potential. For the EAM potential that we use, the chiral icosahedron at the sizes 127, 279 and the anti-Mackay icosahedron at the sizes 521, 873 are unstable under local relaxation.

**Figure 2:**
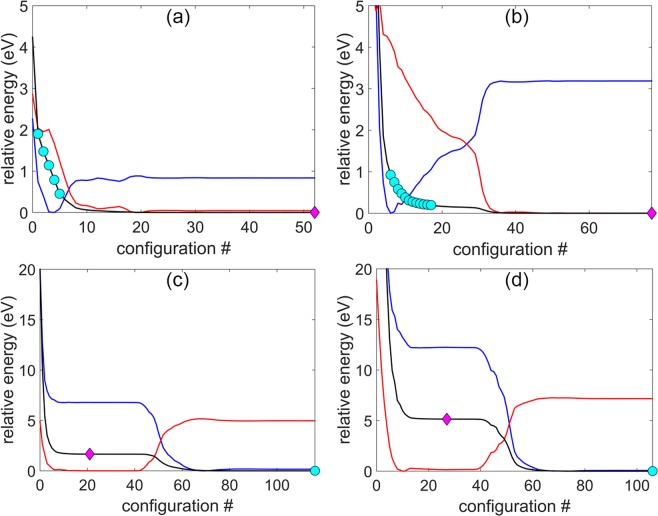
Relative energy of Ag shell (blue), Cu core (red) and whole particle (black) during relaxation for (**a**) Ag${}_{72}$Cu${}_{55}$ (**b**) Ag${}_{132}$Cu${}_{147}$ (**c**) Ag${}_{212}$Cu${}_{309}$ and (**d**) Ag${}_{312}$Cu${}_{561}$. The structure at the beginning of relaxation is chiral icosahedron for (**a**,**b**) and anti-Mackay icosahedron for (**c**,**d**). The relative energy is measured with respect to the minimum value of the respective component under consideration. The configurations corresponding to anti-Mackay icosahedron (magenta diamond) and chiral icosahedron (cyan circle) used for the analysis of core, shell energy are marked in each plot.

Consider the energy variation of the individual components (core, shell) during the relaxation from a higher energy structure i.e. for the sizes of 127, 279 the initial structure for relaxation is a chiral icosahedron and that for the sizes 521, 823 is an anti-Mackay icosahedron. The relaxation process, which is MD at 0 K, is described in the Methods section. The initial structures transform to low energy structures (the anti-Mackay icosahedron for the sizes 127, 279 and the chiral icosahedron for the sizes 521, 873) upon relaxation. The plots of the relative energy (measured with respect to the minimum value of the respective components) of the core, the shell and the whole structure during the relaxation are shown in the Fig. 2 for the sizes 127, 279, 521 and 873. We want to compare the energies of the core and the shell in the chiral stacking and the anti-Mackay stacking. For this, representative structures for the chiral stacking at the sizes 127, 279 and representative structures for the anti-Mackay stacking at the sizes 521, 873 need to be defined as they are locally unstable. The plateau region at the beginning of relaxation observed at the sizes 521, 873 (Fig. 2c,d) corresponds to the anti-Mackay structure. We remark that the plateau region is only approximately flat and does not imply that the anti-Mackay icosahedron is locally stable under relaxation. A configuration near the central region of this plateau was chosen to represent the anti-Mackay structure (indicated by a diamond marker) at the sizes 521, 873. The final configuration at the end of the relaxation for the sizes 127, 279 represents the anti-Mackay structure at these sizes.

Unlike for the sizes 521, 873 there is no such plateau for the sizes 127, 279 at the beginning of the relaxation and hence it is not trivial to choose a configuration that would represent the chiral structure. In this case we choose multiple representative chiral configurations instead. The maximum bond length of the new Ag-Ag bonds for the size 873 is 3.03 Å and that for the size 521 is 3.16 Å. The new Ag-Ag bonds become more stretched with decreasing size which was also reported by Bochicchio *et al*.^20^ Based on this, the multiple chiral configurations were chosen such that the maximum bond length of the new Ag-Ag bonds (such as the black lines in the Fig. 1) in these configurations would fall in the range of 3.00 Å − 3.30 Å for the size 279 and in the range of 3.00 Å − 3.50 Å for the size 127. The configurations that represent the chiral structures are indicated by a circle markers in the Fig. 2. The multiple chiral structures chosen for the sizes 127 and 279 are shown in Figs. S1 and S2 in the Supplementary Information where the chiral stacking of the Ag shell is clearly visible. The representative anti-Mackay structures for the sizes 521 and 873 are shown in the Fig. S3 in the Supplementary Information. In the next section we will discuss about the energy differences between the chiral stacking and the anti-Mackay stacking observed at 50 K for the size 279 which supports the choice of these multiple chiral configurations.

The energy of the Ag shell in the chiral icosahedron is lower in comparison to the Ag shell in the anti-Mackay icosahedron at all sizes irrespective of the stability order. Similarly, the energy of the Cu core in the chiral icosahedron is higher in comparison to the Ag shell in the anti-Mackay icosahedron at all sizes. This implies that at any given size, whether the low energy structure is anti-Mackay or chiral, the energy of the Ag shell is lowered on transition to the chiral structure. The Cu core exhibits an opposite trend, its energy increases in the chiral structure. The balance between these two opposing factors dictates the stability of the anti-Mackay and the chiral structures. We define the following terms which will be used to assess the stability of the anti-Mackay (aM) and the chiral motifs. The energy of the whole structure is the sum of the energy of the Ag shell and the energy of the Cu core.

1 $${E}_{chiral}={E}_{Ag-shell,chiral}+{E}_{Cu-core,chiral}$$

2 $${E}_{aM}={E}_{Ag-shell,aM}+{E}_{Cu-core,aM}$$

3 $${\Delta }_{Ag-shell}={E}_{Ag-shell,chiral}-{E}_{Ag-shell,aM}$$

4 $${\Delta }_{Cu-core}={E}_{Cu-core,chiral}-{E}_{Cu-core,aM}$$

5 $${\Delta }_{total}={E}_{chiral}-{E}_{aM}={\Delta }_{Ag-shell}+{\Delta }_{Cu-core}$$

**Figure 3:**
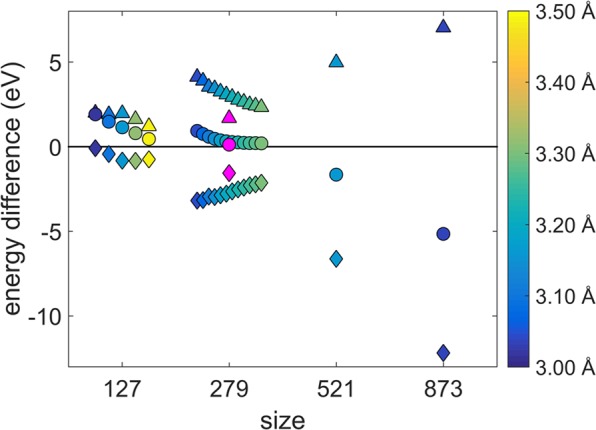
Plot of $\Delta$ parameters as a function of size. ${\Delta }_{Ag-shell}$, ${\Delta }_{Cu-core}$, and ${\Delta }_{total}$ are indicated by diamond, triangle and circle markers respectively. The $\Delta$ parameters at sizes 127 and 279 are shown for the same multiple chiral structures as indicated in the Fig. 2. The color coding indicates the maximum bond length of the new Ag-Ag bonds in the chiral structure at each data point. There is an additional data point marked in magenta color which corresponds to the mean energy differences in the shell (diamond marker), the core (triangle marker), and the total structure (circle marker) observed in the chiral stacking with respect to the anti-Mackay stacking at 50 K.

The $\Delta$ parameters in the above equations measure the energy of a component (core, shell) or the whole structure (total) in the chiral icosahedron with respect to the anti-Mackay icosahedron. ${\Delta }_{total}$ is the relative energy of the chiral icosahedron with respect to the anti-Mackay icosahedron. If ${\Delta }_{total} > 0$, then the anti-Mackay icosahedron is stable and when ${\Delta }_{total} < 0$, the chiral icosahedron is stable. The $\Delta$ parameters are plotted as a function of the nanoalloy size in the Fig. 3. For the sizes 127, 279 the multiple data points correspond to the multiple chiral structures indicated in the Fig. 2. It is clear that the Ag shell energy is lower in the chiral stacking compared to the anti-Mackay stacking while the Cu core has higher energy in the chiral stacking. This shows that the core and the shell prefer different stacking and the balance between these two opposing preferences explains the stability of a particular stacking at each size. For the sizes 127 and 279, the decrease in the energy of the Ag shell is smaller than the increase in the energy of the Cu core. As a result, ${\Delta }_{total}$ is positive and the anti-Mackay stacking is energetically favourable for the whole structure at these sizes. In contrast, for the sizes 521 and 873, the decrease in the energy of the Ag shell is larger than the increase in the energy of the Cu core resulting in negative ${\Delta }_{total}$. Hence, the chiral stacking is energetically favourable at the sizes 521 and 873.

### Thermally induced transition to chiral stacking

**Figure 4:**
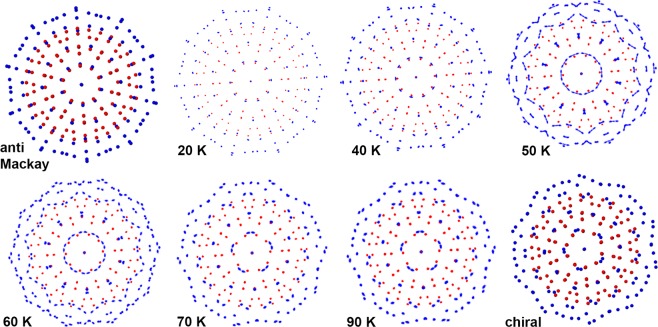
Structure of Ag${}_{132}$Cu${}_{147}$ as a function of temperature (Ag in blue, Cu in red). The images labelled anti Mackay, chiral are for the perfect anti-Mackay, chiral icosahedron. Chiral stacking begins to appear at 50 K.

In this section, we will look at the effect of temperature on the anti-Mackay stacking. The structural changes of 279-atom nanoalloy in the temperature range 20 K − 90 K is shown in the Fig. 4. At any given temperature, the image in the Fig. 4 shows the projection along a 5-fold axis. This image is generated by plotting all the positions visited by an atom every 2.5 ps while the temperature is held constant for 2.5 ns. The positions of the Ag (Cu) atoms are colored in blue (red). For reference, the ideal anti-Mackay and the chiral structures have also been included in the figure. At the size of 279, the anti-Mackay icosahedron is energetically favourable and this structure is maintained at 20 K and 40 K. At 70 K and above, the nanoalloy adopts the chiral icosahedron structure. This indicates that there has been a transition to the chiral stacking from the anti-Mackay stacking upon heating. At the transition temperature (~50 K), all the three structures (anti-Mackay icosahedron, chiral icosahedron with either clockwise or anti-clockwise rotation of the {111}-like planes) co-exist. At 60 K, the anti-Mackay icosahedron is no longer observed, but, the chiral icosahedron with both the clockwise and the anti-clockwise rotations are observed.

**Figure 5:**
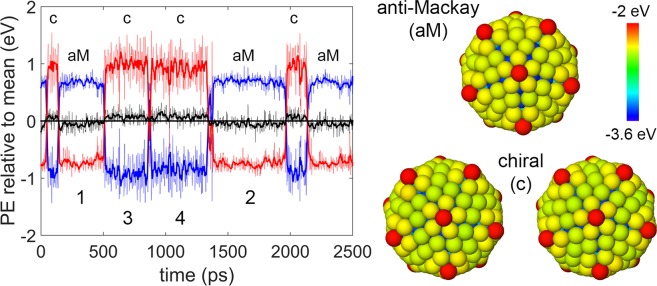
Plot of potential energy (PE) of Ag shell (blue), Cu core (red) and whole structure (black) during heating of Ag${}_{132}$Cu${}_{147}$ at 50 K for 2500 ps. The energy is measured relative to mean of the respective component. The solid lines indicate the energy values after smoothing of the actual energy data (dotted lines). Anti-Mackay, chiral icosahedron structures are indicated by *aM*, *c* respectively. Also included in this figure are the structures of the nanoalloy with anti-Mackay (aM) stacking and the chiral (**c**) stacking observed during the heating. The chiral stacking can have either clockwise or anti-clockwise rotation of the {111}-like planes.

The time evolution of the structural changes can be studied by observing the variation in the energy (relative to the mean) for the Ag shell, the Cu core and the whole structure as a function of time. Such a plot is shown in the Fig. 5 for the nanoalloy Ag${}_{132}$Cu${}_{147}$ at 50 K where the structures with both the chiral stacking and the anti-Mackay stacking co-exist. The regions where the Ag shell (Cu core) energy is lower (higher) correspond to the chiral structure. It is relatively easier to identify the structural transitions based on the energies of the core and the shell compared to the total energy. The nanoalloy transitions between the chiral and the anti-Mackay stacking which are marked as ‘c’ and ‘aM’ respectively in the Fig. 5. Snapshots of the the anti-Mackay stacking and the chiral stacking observed during the heating are also shown in the Fig. 5. The chiral stacking can have either clockwise or anti-clockwise rotation of the {111}-like planes. The chiral structure is still higher in energy compared to the anti-Mackay icosahedron. The mean energy difference between the chiral stacking and the anti-Mackay stacking is ~0.12 eV. This was calculated by considering the regions marked ‘1’, ‘2’ in the Fig. 5 to represent the anti-Mackay stacking and the regions marked ‘3’, ‘4’ to represent the chiral stacking. The $\Delta$ parameters corresponding to this are also plotted in magenta color in the Fig. 3. Referring to the Fig. 3, the $\Delta$ parameters calculated using the multiple representative chiral configurations are close to the $\Delta$ parameters calculated at 50 K based on the heating simulations. This shows that, although the chiral configurations are unstable under local relaxation, the chosen multiple chiral configurations provide reasonable representative chiral configurations for comparing the core and the shell energies in the chiral and the anti-Mackay stacking.

**Figure 6:**
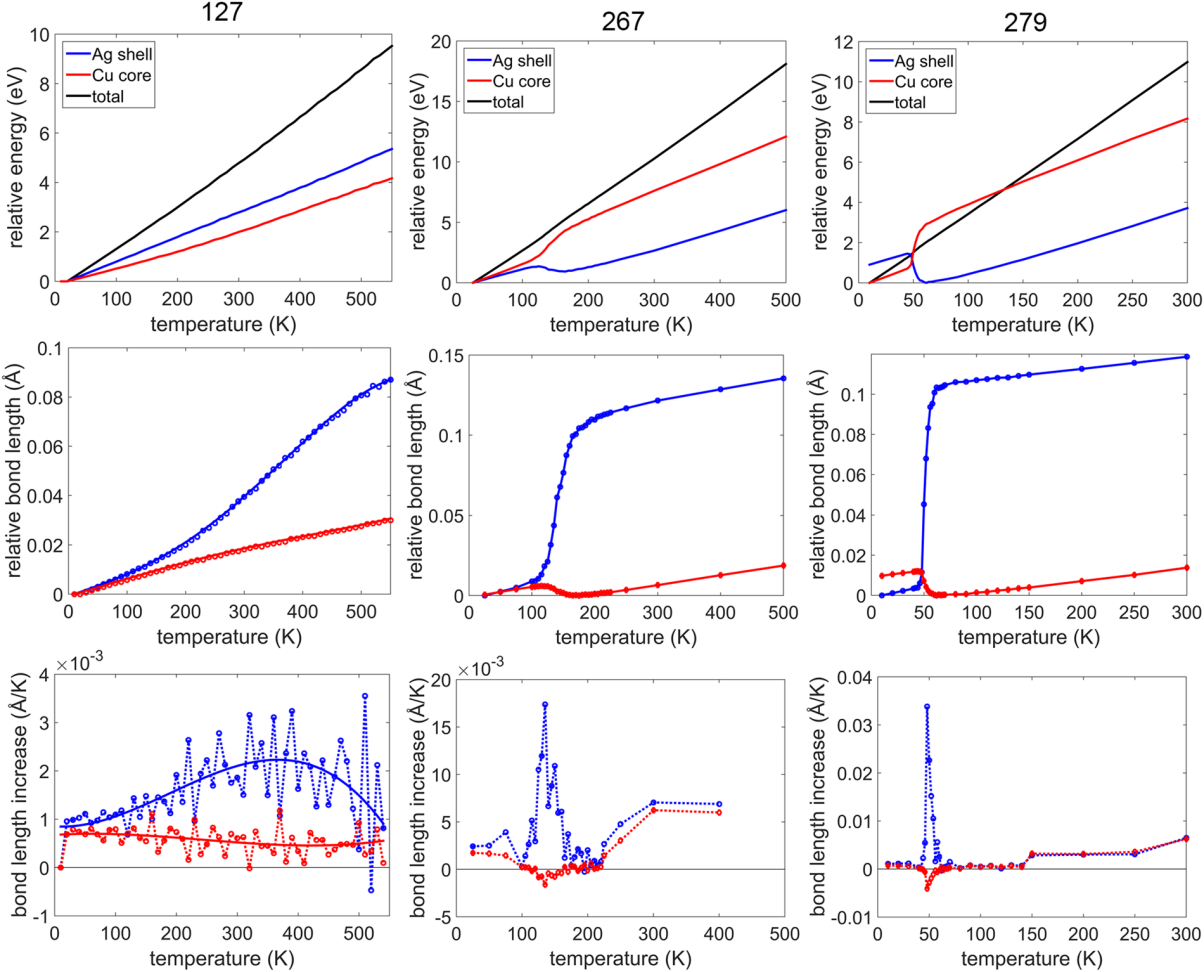
Top row: energy (measured relative to the minimum value) of Ag shell (blue), Cu core (red) and whole structure (black) as function of temperature. middle row: average bond length (relative to the minimum value) of Ag-Ag bonds on shell (blue) and Cu-Cu bonds (red) as a function of temperature. bottom row: rate of change of average bond length plots shown in the middle row. The rate of change of average bond length for 127 (solid line) is based on polynomial fit of the relative bond length.

Temperature dependent transition to the chiral stacking occurs at the sizes of 267 and 127 as well. The size 267 refers to the nanoalloy Ag${}_{120}$Cu${}_{147}$ which has a complete Ag shell with 12 missing vertices. To study the effect of temperature on the chiral stacking, we look at the shell and the core energies as a function of temperature. This is shown for the sizes 127, 267, 279 in the top row of the Fig. 6. By considering the shell and the core energies the temperature dependent transition can be clearly identified at the sizes 279 and 267 but not at the size 127. It was observed that there is a slight compression of the Cu core along with the Ag shell expansion on transition to the chiral stacking which was also reported in an earlier work.^21^ Hence, we use the change in the average bond length of the Ag-Ag bonds of the shell and the Cu-Cu bonds of the core as the quantitative parameters to study the temperature dependent transition to the chiral stacking. These are shown in the middle row of the Fig. 6 for the sizes 127, 267, and 279. The cutoff distance used for identifying the Ag-Ag (the Cu-Cu) bonds is 3.5 Å (3.0 Å). These cutoffs were chosen to include the new Ag-Ag bonds on the surface in the chiral stacking. At the onset of the transition to the chiral stacking, the average Ag-Ag bond length of the Ag shell increases along with a decrease in the average Cu-Cu bond length of the Cu core. Unlike the energy of the Ag shell for the size 127, there is a noticeable change of slope in the average Ag-Ag bond length. The rate of change of the bond length (defined as the change in the average bond length per temperature interval) is shown in the bottom row of the Fig. 6 for the sizes 127, 267 and 279. The rate of change of the average bond length for the size 127 is calculated from a polynomial fit of the relative bond length. During the transition, the rate of change of the average bond length increases for the Ag-Ag bonds on the shell and decreases (slightly in comparison to the Ag-Ag bonds) for the Cu-Cu bonds in the core. The range of the temperature over which the transition to the chiral stacking occurs increases as the size of the nanoalloy decreases from 279 to 127. The range of temperature over which the transition occurs for 267, 279 is approximately 100 K - 200 K, 40 K - 60 K respectively.

**Figure 7:**
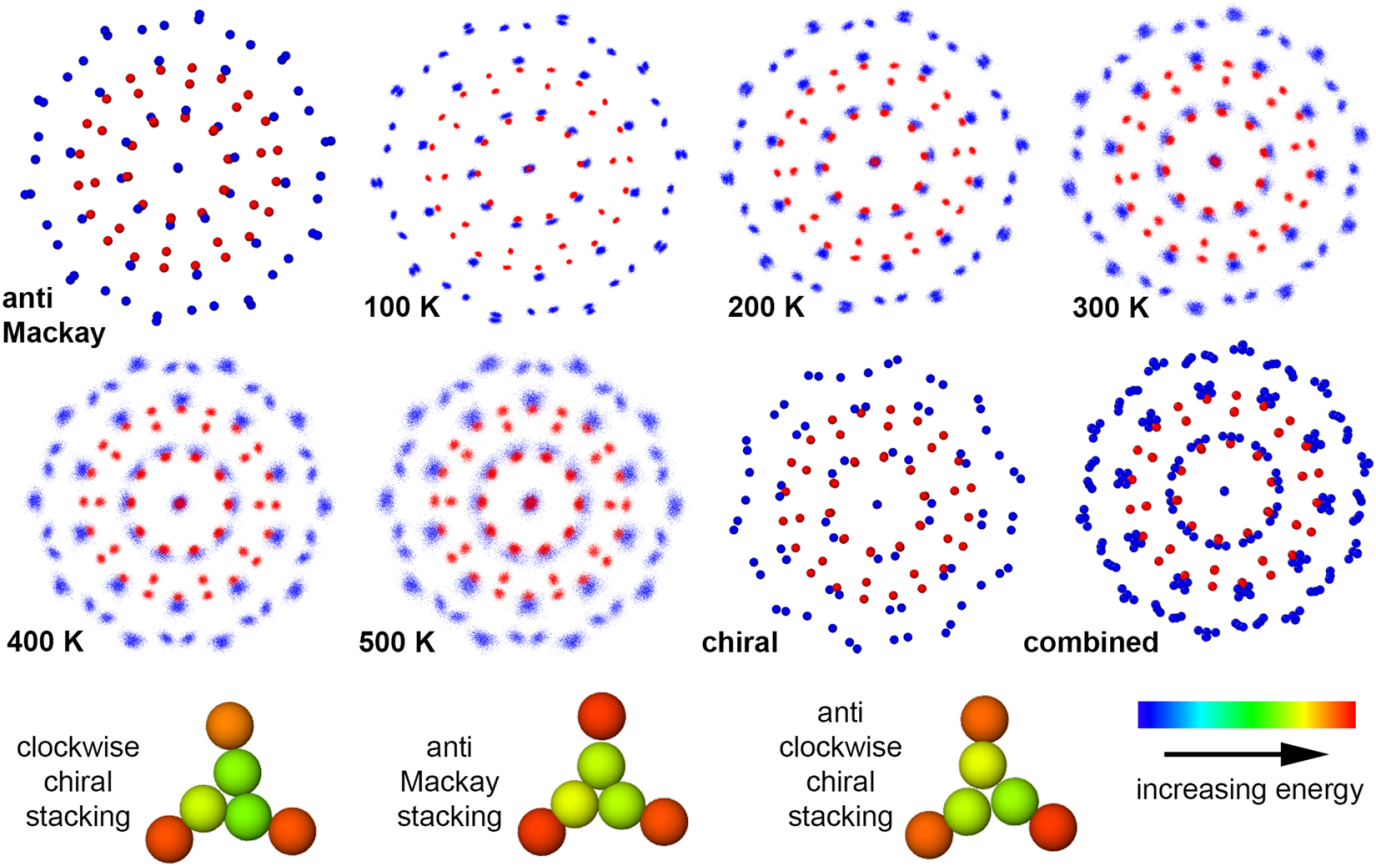
Structure of Ag${}_{72}$Cu${}_{55}$ as a function of temperature (Ag in blue, Cu in red). The images labelled anti Mackay, chiral, combined are for the perfect anti-Mackay, chiral icosahedron and a structure with atomic positions corresponding to anti-Mackay, clockwise and anti-clockwise chiral stacking. bottom row: the three different types of stacking that a {111}-like plane can adopt (atoms belonging to the {111}-like plane coloured in yellow-green).

At the size of 127, the transition occurs over a much wider temperature range compared to the sizes 267, 279. This is evident from the broad peak in the rate of change of the Ag-Ag bond length accompanied by a slight decrease in the rate of change of the Cu-Cu bond length. In the structures with the chiral stacking at the sizes 267, 279, all the {111}-like planes show a concerted rotation. In contrast to this, only few {111}-like planes in the chiral structures at the size 127 exhibit concerted rotation. This can be better understood by observing the structures at different temperatures (similar to 279-atom nanoalloy in Fig. 4). The structures are shown in the temperature range 100 K - 500 K in the Fig. 7. Also included in the figure are the ideal structures corresponding to the anti-Mackay, the chiral icosahedron and a combined structure (where the atomic positions correspond to the anti-Mackay, the clockwise and the anti-clockwise chiral stacking) for reference. The structures at 100 K, 200 K closely resemble the anti-Mackay stacking. At higher temperatures, a clear chiral stacking cannot be observed. For the size 279, at the temperatures where both the chiral stacking and the anti-Mackay stacking are observed, at any given instant of time, we observe either completely the chiral stacking or the anti-Mackay stacking. In contrast, for the size 127, complete chiral stacking is not observed. The chiral stacking almost always occurs only partially i.e. both the chiral stacking and the anti-Mackay stacking are observed in the same configuration (see Fig. S4 in the Supplementary Information for an example). However, complete anti-Mackay stacking can be observed. As a result when we look at the cumulative structure over a time interval at a specific temperature (such as the images in the Fig. 7), it resembles the combined structure shown in the Fig. 7. The structures at 300 K, 400 K and 500 K clearly resemble the combined structure. The extent of chiral stacking increases from 300 K to 500 K which can also be observed from the increase in the average Ag-Ag bond length with increasing temperature in the Fig. 6 for the size 127. The {111}-like planes can adopt three possible stacking configurations: anti-Mackay, clockwise or anti-clockwise chiral stacking (bottom row of Fig. 7).

### Anti-Mackay, chiral stacking in nanoalloys with incomplete shells

**Figure 8:**
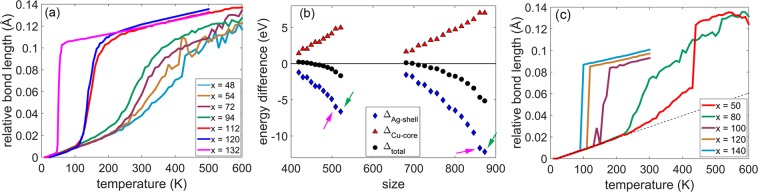
(**a**) Average bond length (relative to the minimum value) of Ag-Ag bonds on shell as a function of temperature in Ag${}_{x}$Cu${}_{147}$ with varying number of Ag atoms (*x* in the range 48 - 132). $x=132$ represents the perfect Ag${}_{132}$Cu${}_{147}$ with complete shell. (**b**) Plot of $\Delta$ parameters with progressive removal of {111}-like planes on the surface from structure with 12 missing vertices (indicated by magenta arrow). The perfect structures with complete shells are indicated by green arrows for Ag${}_{212}$Cu${}_{309}$, Ag${}_{312}$Cu${}_{561}$. (**c**) average bond length (relative to the minimum value) of Ag-Ag bonds on shell as a function of temperature in Ag${}_{x}$Cu${}_{309}$ with varying number of Ag atoms (*x* in the range 50 - 140).

Till now, the structures with complete shells and 12 missing vertices in the case of Ag${}_{120}$Cu${}_{147}$ have been considered. We now look at the structures with incomplete shells. Nanoalloys having a Cu core with 147 atoms are designated as Ag${}_{x}$Cu${}_{147}$ (*x* is the number of Ag atoms in the shell). The shell is complete when *x* is 132 or 120 (with 12 missing vertices). The average Ag-Ag bond length of the shell is shown as a function of temperature for varying number of Ag atoms in the shell in the Fig. 8a with *x* in the range 48 - 132. These structures were constructed based on the optimal structures identified in the work by Bochicchio *et al*.^21^ and are shown in the Fig. S5 in the Supplementary Information. The transition temperature and the range of the temperature over which the transition occurs both increase with decreasing number of Ag atoms in the shell. However, there are three distinct regions based on the number of Ag atoms in the shell (*x*): *x* = 132; *x* = 120, 112; and *x* = 94, 72, 54, 48. In these regions, the transition occurs in the range 40 K - 60 K, 100 K - 200 K, 200 K - 450 K. This shows that, for a fixed size of the core, the tendency for chiral stacking is stronger for complete shells compared to incomplete shells.

This can be understood by considering Ag${}_{200}$Cu${}_{309}$, Ag${}_{300}$Cu${}_{561}$ nanoalloys (with 12 missing vertices) and progressively removing the {111}-like planes (one-by-one) from the complete shell. For Ag${}_{200}$Cu${}_{309}$, up to nine {111}-like planes were removed. In case of Ag${}_{300}$Cu${}_{561}$, up to twelve {111}-like planes were removed. Removing an additional {111}-like plane renders the chiral structure unstable under local relaxation. The structures constructed in this manner do not necessarily represent the global minimum. Since the motivation is to understand the preference for chiral stacking with varying number of Ag atoms in an incomplete shell, the structures constructed in this manner would be sufficient. The plot of $\Delta$ parameters for these structures is shown in the Fig. 8b. As the number of Ag atoms in the shell decreases, the $\Delta$ values for the Ag shell and the Cu core approach zero. But the energy of the Ag shell (the Cu core) in the chiral stacking remains lower (higher) in comparison to the anti-Mackay stacking. This indicates that the differences in the core and the shell energies between the anti-Mackay and the chiral stacking are lowered as the shell becomes progressively incomplete with lesser number of Ag atoms in the shell. As a result, there is a transition to anti-Mackay stacking when 6 (11) {111}-like planes are removed from Ag${}_{200}$Cu${}_{309}$ (Ag${}_{300}$Cu${}_{561}$). This is consistent with the results reported by Bochicchio *et al*.^21^ where the global minimum for 84 Ag atoms on 309-atom Cu core had anti-Mackay stacking.

Thermally induced transition is also observed for the structures with 309-atom, 561-atom Cu cores where the incomplete Ag shells prefer anti-Mackay stacking. The Ag shells on a 309-atom Cu core prefer anti-Mackay stacking when at least 6 {111}-like planes are removed from the complete shell Ag${}_{200}$Cu${}_{309}$ nanoalloy (${\Delta }_{total}$ becomes positive, see Fig. the 8b). The average Ag-Ag bond length of the shell of Ag${}_{x}$Cu${}_{309}$ is shown as a function of temperature in the Fig. 8c. The values of *x* considered are 50, 80, 100, 120, 140 which correspond to 15, 12, 10, 8, 6 {111}-like planes removed. The transition to the chiral stacking occurs in a relatively narrow range for incomplete shells with 140, 120, 100 Ag atoms. When the number of Ag atoms in the incomplete shell decreases to 80 or 50, the transition to chiral stacking occurs over a broader range of temperature. The sharp increase in Ag-Ag bond length at around 430 K when there are 50 Ag atoms on the shell indicates the rearrangement of the shell with a mix of Mackay stacking (fcc-like stacking), anti-Mackay stacking and chiral stacking. The above analysis results in two main observations. (i) The differences in the core and the shell energies across the anti-Mackay and the chiral stacking reduce as the Ag shell becomes progressively incomplete with lesser number of atoms. This results in a transition to the anti-Mackay stacking below a certain number of Ag atoms in the shell. (ii) Upon heating, the shells in these structures adopt chiral stacking.

## Discussion

**Figure 9:**
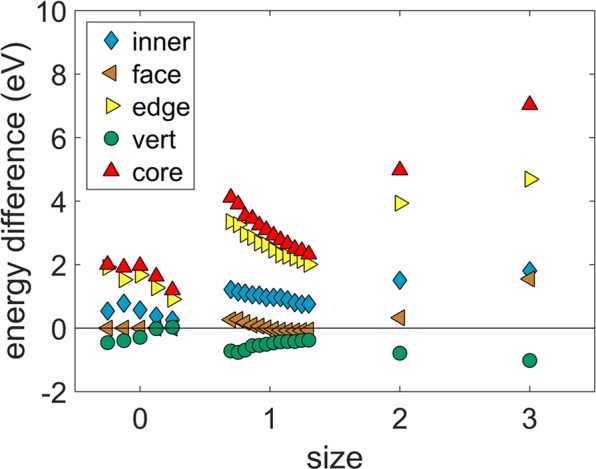
Contribution of various components to ${\Delta }_{Cu-core}$. The core is separated into inner region where the Cu atoms have only Cu atoms as the first nearest neighbors (labelled as *inner*), faces of {111}-like planes in the subsurface Cu layer (labelled as *face*), edges in the subsurface Cu layer (labelled as *edge*), and vertices in the subsurface Cu layer (labelled as *vert*).

Our analysis shows that the interplay between the core and the shell dictates the size dependent stability of the anti-Mackay and the chiral stacking in AgCu nanoalloys. We look at this closely by separating the Cu core into further components. The inner region of the Cu core excluding the subsurface Cu layer can be treated as one component. The subsurface Cu layer can be further divided into three components: faces of the {111}-like planes, the edges and the vertices. The contribution of these distinct components to the $\Delta$ parameter of the Cu core is shown in the Fig. 9. We can readily see that the Cu atoms present in the edges of the subsurface layer result in a relatively higher amount of energy increase on transition to the chiral stacking compared to the other components. With increase in the size, the relative contribution of the edges decreases and those of the inner region and the faces increases. Vertices show an opposite behaviour, in that, their energy decreases on transition to the chiral stacking.

Although most of the energy increase in the core on transition to the chiral stacking is due to the subsurface Cu atoms on the edge, the other components (inner region, faces and vertices) also contribute significantly to the overall increase in the core energy. Moving onto the Ag atoms in the shell, except at the size 127, all Ag atoms in the shell show a decrease in energy on transition to the chiral stacking (see Fig. S6 in the Supplementary Information). Consider the size 873, three atoms at the center of the {111}-like planes do not form any new Ag-Ag bonds and they are slightly displaced from the ideal hcp-like stacking positions. However, their energy also decreases on transition to the chiral stacking (see Fig. S7 in the Supplementary Information). This shows that there could be additional factor which plays a role in stability of anti-Mackay, chiral stacking along with the simple bond counting argument^20^ which proposed that the chiral stacking becomes stable as the energy gain due to the formation of new Ag-Ag bonds overcompensates the displacement of the Ag atoms from the ideal hcp-like positions. However, referring to the Fig. 3, if we consider only the shell energy, it would appear that the chiral stacking should prevail at all the sizes and not only at the sizes of 521 and 873. But when both the core and the shell energies are considered together, we can see that balance of these two opposing factors results in the chiral stacking being lower in energy at the sizes 521, 873 and the anti-Mackay stacking being lower in energy at the sizes 127, 279.

**Figure 10:**
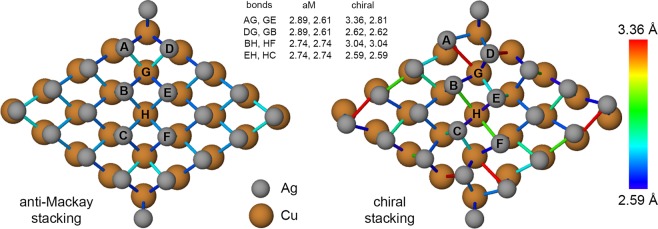
Ag-Cu bonds in anti-Mackay and chiral stacking at the size 521. In the direction which is roughly parallel to the direction where new Ag-Ag bonds are formed on the surface (DB, EC), the Ag-Cu bonds contract and in the perpendicular direction, the Ag-Cu bonds expand. The bond lengths (in Å) of certain Ag-Cu bonds are listed for anti-Mackay (aM) stacking and chiral stacking assuming the bonds are retained in chiral stacking. Ag atoms are drawn with smaller radius to clearly show the subsurface Cu atoms.

Consider the Ag-Cu bonds in the anti-Mackay and the chiral stacking at the size 521 shown in Fig. 10 viewed in the direction of a 2-fold axis. Only two {111}-like planes are shown on the surface and subsurface layers. On transition to the chiral stacking, new Ag-Ag bonds are formed between the atoms B,D and the atoms C,E. As a result, in the direction which is roughly parallel to BD and CE, the Ag-Cu bonds (DG, GB and EH, HC) become smaller (see the values of the bond lengths listed in the Fig. 10). Concurrently, in the perpendicular direction, the Ag-Cu bonds (AG, GE and BH, HF) become larger. As a consequence, some of the Ag-Cu bonds become stretched and some become smaller on transition to the chiral stacking. At the same time, even without considering the new Ag-Ag bonds on the surface, the other Ag-Ag bonds become slightly stretched. Also, the Cu-Cu bonds are slightly compressed. Given the bond length changes of the Ag-Cu, the Ag-Ag, and the Cu-Cu bonds, it would be interesting to see the implication of this (if any) in terms of the differences between the electron densities in the anti-Mackay stacking and the chiral stacking which can be calculated using first principles methods such as density functional theory (DFT).

The thermally induced transition to the chiral stacking that we have reported here does not account for the quantum effects as we use a classical atom-atom potential. Panizon *et al*.^30^ compared the quantum expression and the classical approximation for the vibrational part of the partition function for studying the solid-solid transitions in Pd-Pt nanoalloys. They showed that the transition temperatures do not change significantly when quantum effects are considered. Hence, we believe that, although our temperature dependent transition to the chiral stacking is based on classical atom-atom potential, they would remain qualitatively similar when the quantum affects are considered. Panizon *et al*.^30^ also showed that the structures belonging to the same family have different vibrational entropy due to the low frequency modes which involve shearing between the atoms on the surface. The anti-Mackay stacking and the chiral stacking differ only in the surface arrangement. This suggests that the thermal stability of the chiral stacking could be due to the higher vibration entropy of the chiral stacking due to the low frequency modes. This requires further investigation. As the total energy difference between the anti-Mackay stacking and the chiral stacking (${\Delta }_{total}$) increases with decrease in the size of the nanoalloy, the effect of temperature “weakens” with regards to the concerted rotation. At the size of 127, there is no concerted rotation of all the {111}-like planes, instead only few of them adopt chiral stacking at any given instant of time. As a result, on heating, the structure has combined features of both anti-Mackay stacking and chiral stacking as shown in the Fig. S4 in the Supplementary Information.

Bimetallic nanoalloys are synthesized either in gas phase or in solution^1,2,31^. However, these methods may not result in equilibrium structures due to the non-equilibrium processes and the kinetics of the synthesis procedure^8,32^. One possible method through which we may verify the results of the current work experimentally is to coat the AgCu nanoalloys of desired size with silica in a colloidal solution as is done for monometallic particles^33,34^. Silica shell acts like an inert barrier that ensures that the size of nanoalloy is maintained. The core-shell structures may then be subjected to *in situ* heating in a transmission electron microscope (TEM) to form structures close to equilibrium. This allows us to verify the size-dependent chiral stacking and thermally induced transition to chiral stacking. The current work was focused on AgCu nanoalloys. The chiral stacking was predicted for AgCo, AgNi and AuNi nanoalloys as well. Given that these systems share common features with AgCu alloys, it would be interesting to see if the core-shell interplay is qualitatively similar or different in these systems compared to AgCu.

## Conclusions

In summary, our results show that the interplay between the Ag shell and the Cu core of AgCu nanoalloys dictates the size-dependent stability of chiral stacking. The Ag shell (Cu core) has lower (higher) energy in the chiral stacking compared to the anti-Mackay stacking at all sizes considered in the current work. However, if we consider the shell energy alone, it can be inferred that the chiral stacking is energetically favourable at all the sizes. The Ag shell and the Cu core have opposing preferences. Hence, the energy of Ag shell alone is not sufficient to explain the stability of the chiral stacking. The balance between the core and the shell energies results in anti-Mackay Ag shells for Ag${}_{72}$Cu${}_{55}$, Ag${}_{132}$Cu${}_{147}$ nanoalloys and chiral Ag shells for Ag${}_{212}$Cu${}_{309}$, Ag${}_{312}$Cu${}_{561}$ nanoalloys. At the sizes of 127 and 279 where the anti-Mackay stacking is energetically favourable, heating induces a transition to the chiral stacking. The transition to the chiral stacking at the size of 279 occurs at around 50 K and hence at room temperature the structure will have chiral stacking. At the size 127, there is a relatively weaker tendency to adopt chiral stacking. In this case, the {111}-like planes switch between the anti-Mackay and the chiral stacking continuously. Same observations were made with regards to the incomplete shells i.e. the core-shell energetics can explain the stability of the anti-Mackay or the chiral stacking. The thermally induced transition to the chiral stacking was also observed for incomplete shells. The transition to the chiral stacking is associated with bond length changes. The Ag-Ag bonds on the surface expand along with slight compression of the Cu-Cu bonds. In the case of the Ag-Cu bonds, some of them expand and some of them contract.

## Methods

We used an embedded atom method (EAM)^18^ potential developed by Williams *et al*.^27^ to model the interaction between Ag-Ag, Cu-Cu and Ag-Cu atoms. The EAM potential used in the current work correctly predicts the Ag shell stacking for the AgCu nanoalloys as reported by Bochicchio *et al*.^20^ Also, using this EAM potential we reproduced the surface reconstructions in the truncated octahedra and the decahedra in accordance with the results of Panizon *et al*.^23^. Some of the reconstructed structures are shown in the Fig. S8 and the energy difference between the reconstructed and the unreconstructed structures are reported in the Table S1 in the Supplementary Information. This potential was also used to study the energetics of the formation of the AgCu core-shell nanoalloys previously^35^. All the simulations were carried out using LAMMPS^28^ package. We give a brief description of the simulation methods here. The relaxation process used to obtain the results in the Fig. 2 is MD at 0 K using conjugate-gradient energy minimization. The initial structure for the relaxation process at the sizes 127, 279 is a chiral icosahedron and the initial structure at the sizes 521, 873 is an anti-Mackay icosahedron. The initial chiral structures used for relaxation at the sizes 127, 279 were constructed by rotating the {111}-like planes in anti-Mackay stacking by 19.5${}^{\circ }$^20,36^. The heating simulations were carried out in an NVT ensemble with a time step of 5 fs. Close to the temperature where the transition to the chiral stacking occurs, the temperature was incremented in steps of 2 K, 5 K for the sizes 279, 267 respectively. For all other sizes, a uniform temperature increment of 10 K was used. At any given temperature, the system is equilibrated for 500 ps. Then it is held at that temperature for 2500 ps and configurations were collected every 2.5 ps. The potential energy data in the Fig. 5 was treated to smooth the data using a 7-point moving average method. For calculating the average bond length a cutoff of 3.50 Å was used for the Ag-Ag bonds and a cutoff of 3.00 Å was used for the Cu-Cu bonds. At any given temperature, the average Ag-Ag, Cu-Cu bond length was calculated using 1000 configurations which were collected every 2.5 ps.

## Supplementary information

Supplementary Information.
