# Supplementary material for: Role of core-shell energetics on anti-Mackay, chiral stacking in AgCu nanoalloys and thermally induced transition to chiral stacking
Source: Sci Rep. 2020 Feb 24;10:3296. doi: 10.1038/s41598-020-60059-6 (PMC7039915; doi:10.1038/s41598-020-60059-6)
Supplement: Supplementary file 1 — Supplementary Information. [file 41598_2020_60059_MOESM1_ESM.doc]

**Role of core-shell energetics on anti-Mackay, chiral stacking in AgCu nanoalloys and thermally induced transition to chiral stacking**

Manoj Settem and Anand Krishna Kanjarla

Department of Metallurgical and Materials Engineering, Indian Institute of Technology Madras, Chennai, India – 600 036

Supplementary Information


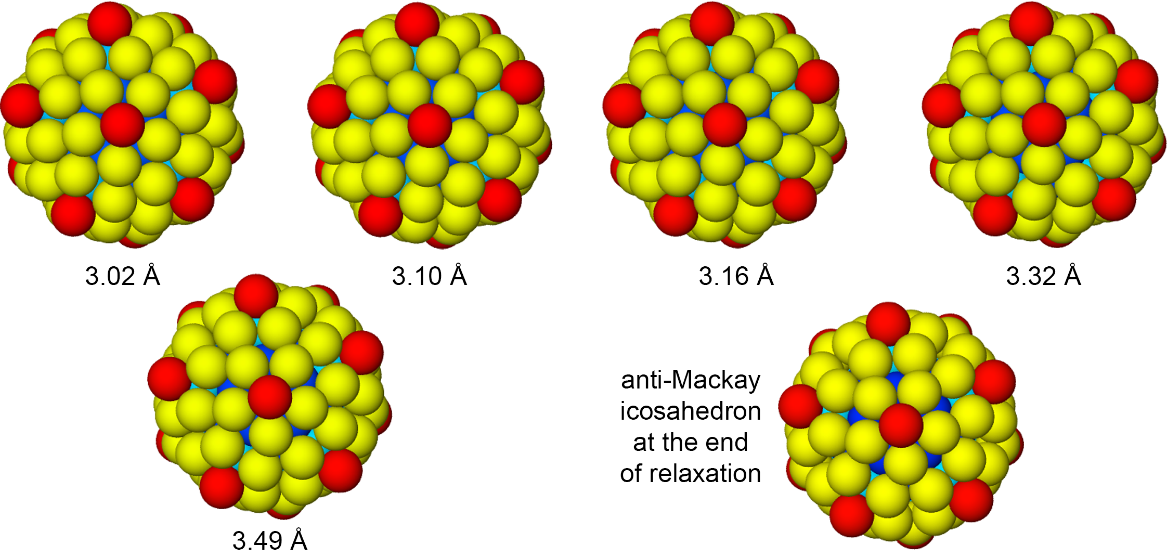


**Figure. S1** The multiple representative chiral configurations along with the maximum bond length of the new Ag-Ag bonds for the size 127. The final anti-Mackay configuration is also shown.


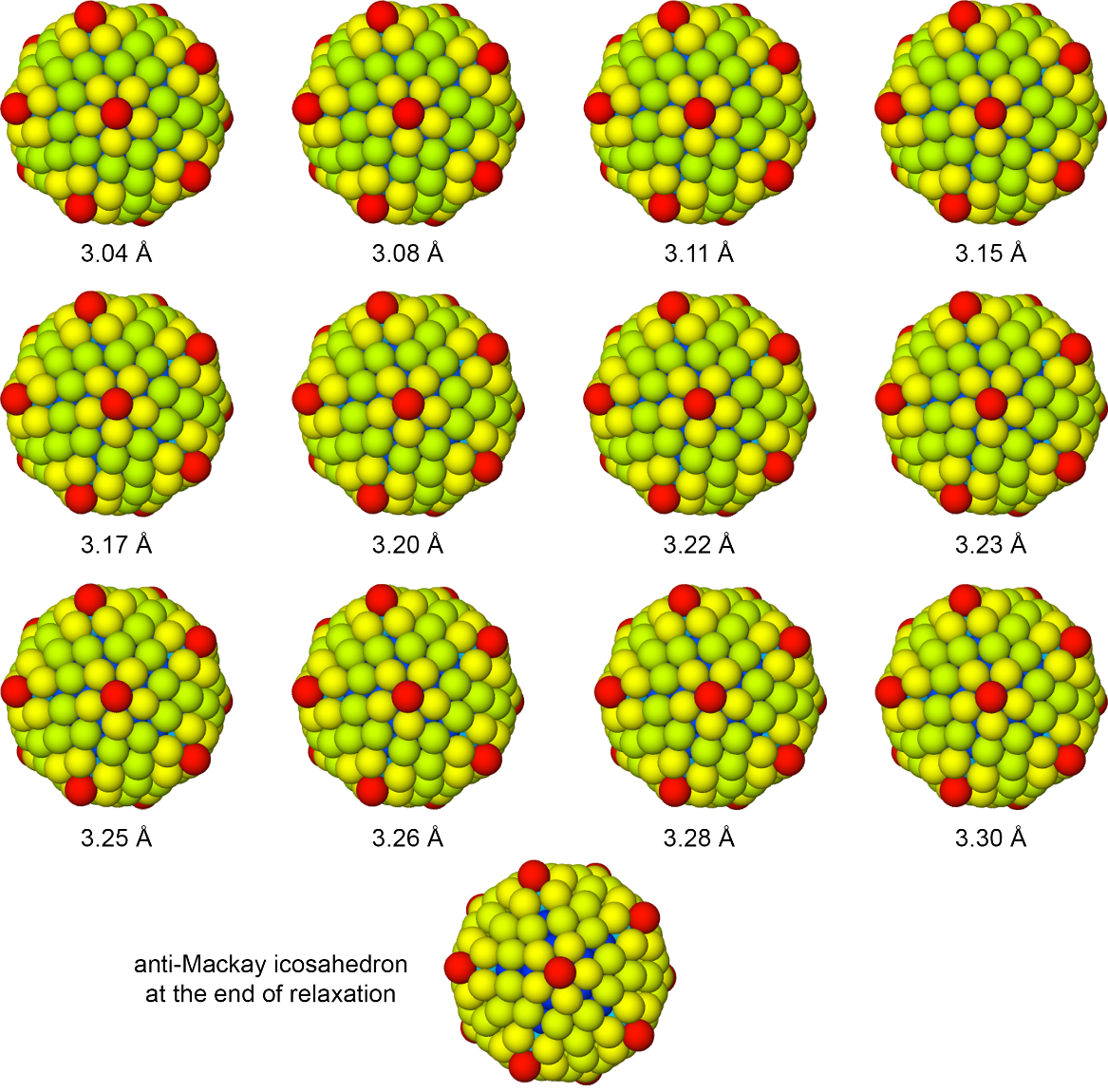


**Figure. S2** The multiple representative chiral configurations along with the maximum bond length of the new Ag-Ag bonds for the size 279. The final anti-Mackay configuration is also shown.


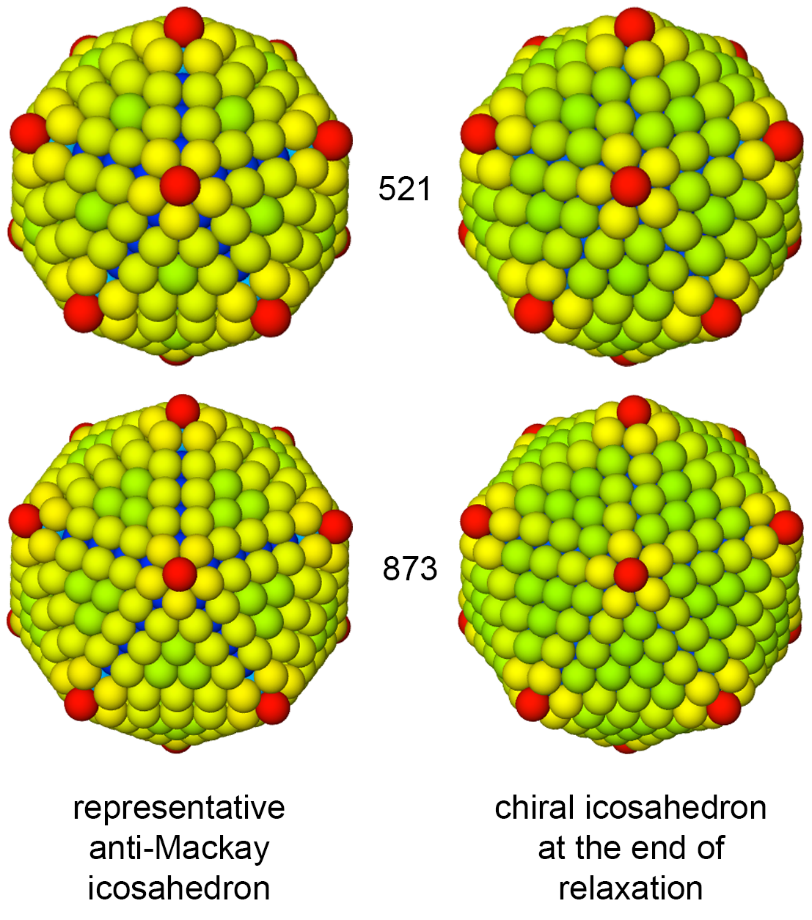


**Figure. S3** The representative anti-Mackay configuration for the sizes 521, 873. The final chiral configuration at these sizes is also shown.


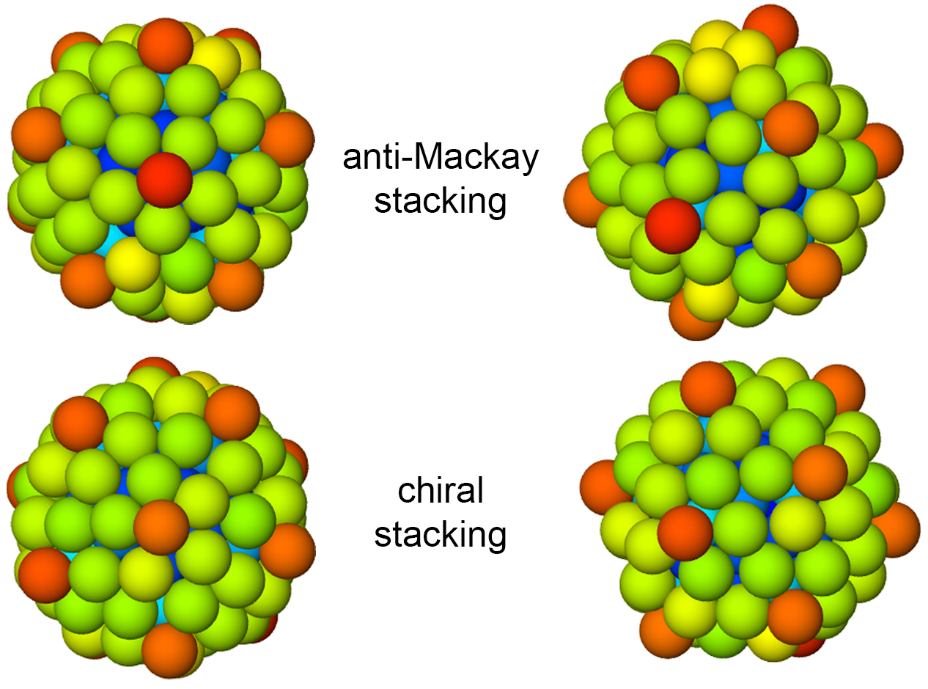


**Figure. S4** An example of chiral stacking and the anti-Mackay stacking co-existing in the same structure during heating simulation for the size 127. The top row shows the anti-Mackay stacking and the bottom row shows the chiral stacking.


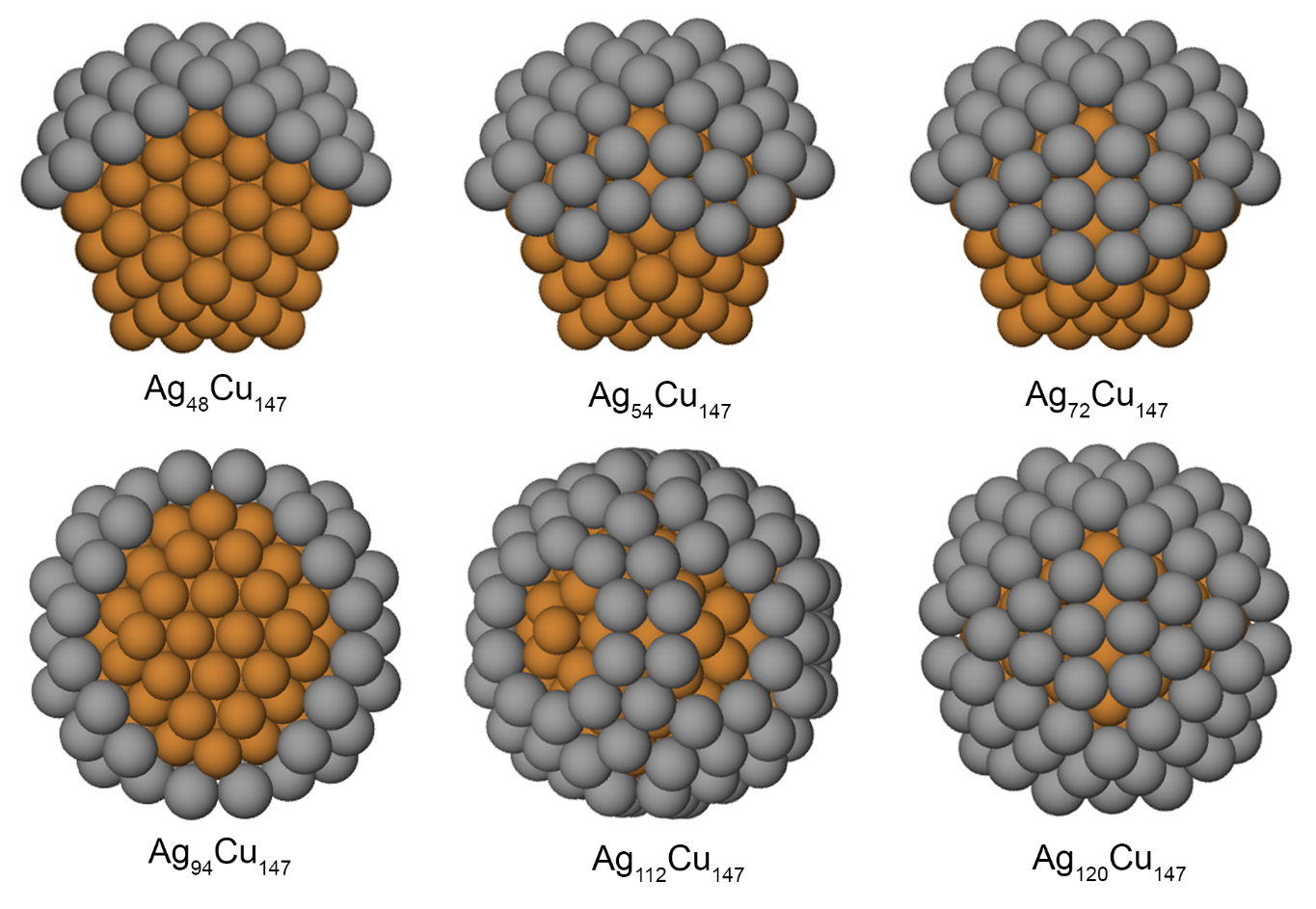


**Figure. S5** Structure of
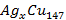
 nanoalloys with incomplete Ag shells. The number of Ag atoms (x) in the shell varies in the range 48 – 120 for these structures.


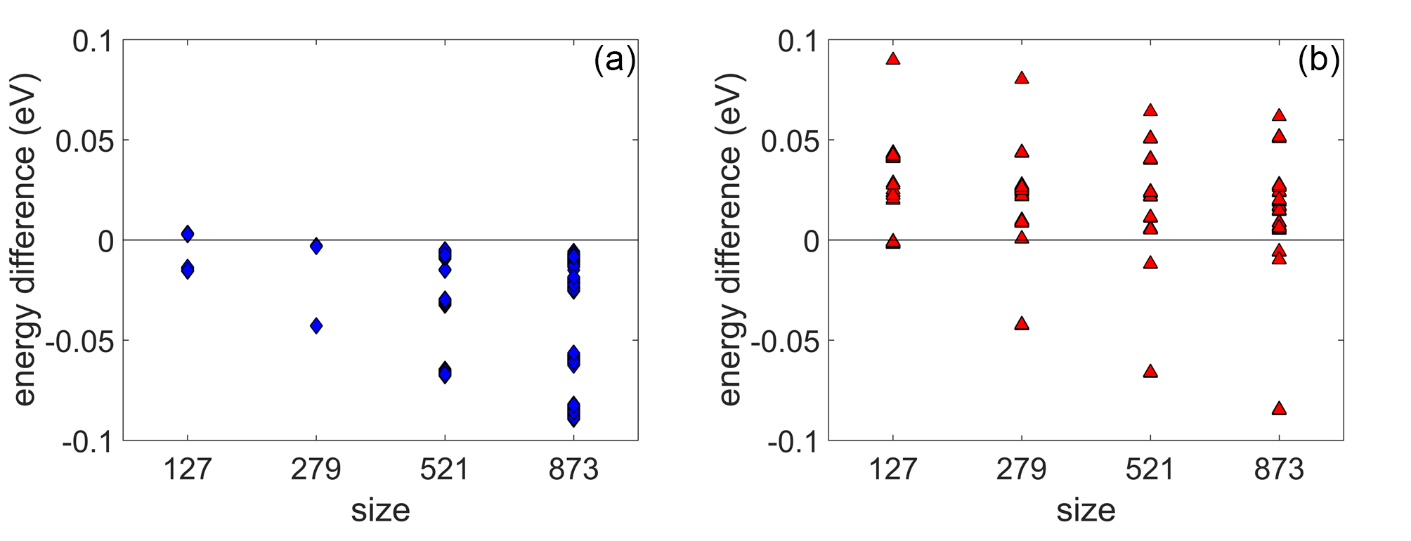


**Figure. S6** Energy of Ag atoms (a) and Cu atoms (b) in the chiral stacking relative to energy in anti-Mackay stacking. Except at the size of 127, the energy of all Ag atoms in the shell have lower energy in chiral stacking.


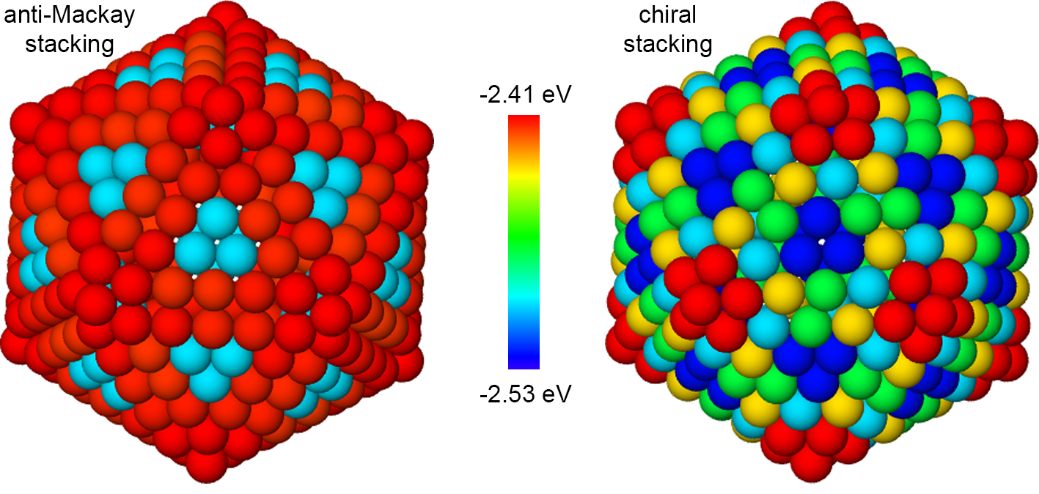


**Figure. S7** Energy of Ag atoms in anti-Mackay stacking and chiral stacking. A very narrow range of energies was chosen for coloring the Ag atoms in order show the decrease of the energy of the three central atoms of the {111}-like planes on transition to chiral stacking. Atoms with higher energies are colored in red and atoms with lower energies are colored in blue.


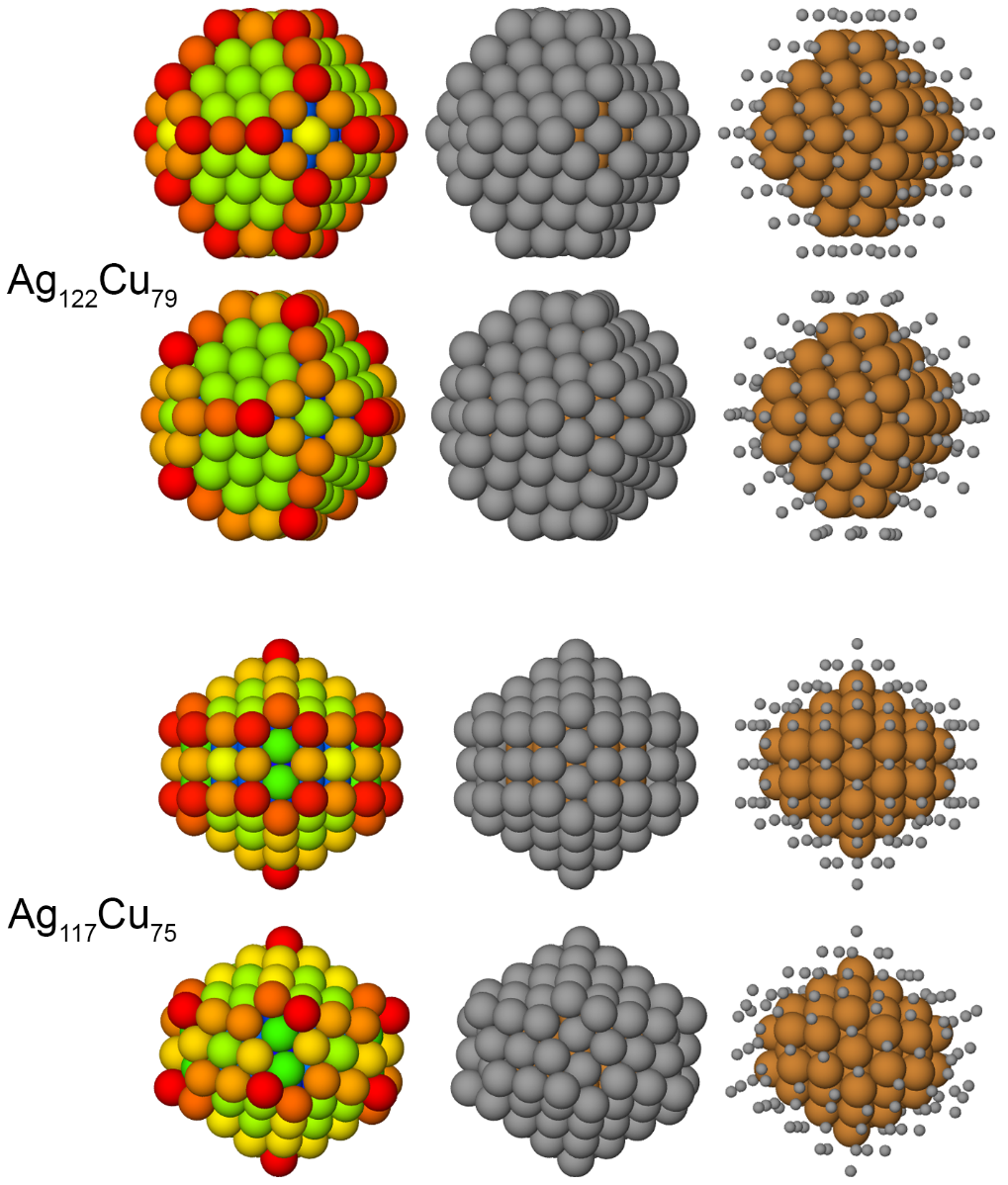


**Figure. S8** For each nanoalloy, the top row shows the unreconstructed structure and the bottom row shows the reconstructed structure. The unreconstructed structures of the nanoalloys
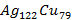
,
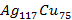
 are truncated octahedron, Marks’ decahedron respectively.

**Table. S1** The energy of the reconstructed structure with respect to the unreconstructed structure (
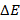
) for the EAM potential used in the current work (
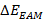
), the second moment approximation to the tight binding (SMTBA) potential (
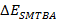
), and based on the density functional theory (DFT) calculations (
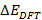
). The values calculated using SMTBA potential and DFT are taken from the reference 23 of the main manuscript.

| Nanoalloy | Unreconstructed structure | 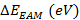 | 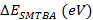 | 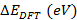 |
| --- | --- | --- | --- | --- |
| 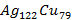 | truncated octahedron | -3.58 | -2.80 | -3.48 |
| 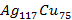 | Marks’ decahedron | -2.67 | -1.73 | -2.64 |
| 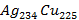 | truncated octahedron | -5.04 |  |  |
| 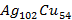 | Ino decahedron | -4.25 |  |  |
